# Restriction of in vivo infection by antifouling coating on urinary catheter with controllable and sustained silver release: a proof of concept study

**DOI:** 10.1186/s12879-018-3296-1

**Published:** 2018-08-06

**Authors:** Kedar Diwakar Mandakhalikar, Rong Wang, Juwita N. Rahmat, Edmund Chiong, Koon Gee Neoh, Paul A. Tambyah

**Affiliations:** 1 Department of Medicine, Yong Loo Lin School of Medicine, National University of Singapore, 1E, Kent Ridge Road, NUHS Tower Block, Level 10, Singapore, 119228 Singapore; 2 ACI Medical Pte Ltd, Singapore, 069534 Singapore; 3 Department of Surgery, Yong Loo Lin School of Medicine, National University of Singapore, Singapore, 119228 Singapore; 4 Department of Chemical and Biomolecular Engineering, National University of Singapore, Singapore, 117585 Singapore

**Keywords:** CAUTI, Silver nanoparticles, Urinary catheter, Mouse model, Porcine model, Catheter associated urinary tract infections

## Abstract

**Background:**

Catheter Associated Urinary Tract Infections are among the most common urological infections world-wide. Bacterial biofilms and encrustation cause significant complications in patients with urinary catheters. The objective of the study is to demonstrate the efficacy and safety of an anti-microbial and anti-encrustation silver nanoparticle (AgNP) coating on silicone urinary catheter in two different animal models.

**Methods:**

Antifouling coating (P3) was prepared with alternate layers of polydopamine and AgNP and an outermost antifouling layer. Sixteen C57BL/6 female mice and two female PWG Micropigs® were used to perform the experiments. In mice, a 5 mm long silicone catheter with or without P3 was transurethrally placed into the urinary bladder. Micropigs were transurethrally implanted – one with P3 silicone catheter and the other with commercially available silver coated silicone catheter. Both models were challenged with *E. coli*. Bacteriuria was evaluated routinely and upon end of study (2 weeks for mice, 3 weeks for micropigs), blood, catheters and bladders were harvested and analysed for bacterial colonization and encrustation as well as for toxicity.

**Results:**

Lower bacterial colonization was seen on P3 catheters as well as in bladders of animals with P3 catheter. Bacteriuria was consistently less in mice with P3 catheter than with uncoated catheters. Encrustation was lower on P3 catheter and in bladder of micropig with P3 catheter. No significant toxicity of P3 was observed in mice or in micropig as compared to controls. The numbers were small in this proof of concept study and technical issues were noted especially with the porcine model.

**Conclusions:**

Antifouling P3 coating reduces bacterial colonization on catheter and in animal bladders without causing any considerable toxicity for 2 to 3 weeks. This novel coating could potentially reduce the complications of indwelling urethral catheters.

**Electronic supplementary material:**

The online version of this article (10.1186/s12879-018-3296-1) contains supplementary material, which is available to authorized users.

## Background

Urinary catheters are among the most common medical devices in clinical use [1]. However, catheter associated urinary tract infections (CAUTI) are among the most common hospital acquired infections [2–4]. The risk of CAUTI is reported to be between 3 and 7% daily and increases with the duration of catheterization [5].

Uropathogenic *Escherichia coli* (UPEC) are responsible for most CAUTI [6]. Many UPEC strains are now resistant to multiple antibiotics, and thus difficult to treat, emphasizing the importance of preventative strategies [7–9].

Intraluminal and extraluminal biofilms, caused by the attachment of bacteria to the catheter, facilitate the entry and persistence of uropathogens in the bladder and thus promote the development of resistance [6]. In addition, urease producing bacteria, especially *P. mirabilis*, contribute significantly to catheter encrustation through precipitation of mineral salts such as calcium phosphate and magnesium ammonium phosphate [10]. These encrustations and biofilms are major contributors to morbidity and mortality in CAUTI [11–14]. They are responsible for the failure of many medical devices [15]. Novel approaches to preventing CAUTI have focussed on preventing the development of biofilms using various techniques including novel coatings [16].

We have previously developed an anti-fouling catheter with controllable and sustained silver release which is effective in resisting encrustation induced by *Proteus mirabilis* in vitro for up to 45 days [17]. The coating comprises multiple layers of silver using polydopamine (PDA) as the surface anchor which reduces the silver ions into silver nanoparticles (AgNPs) and stabilizes and protects the AgNPs from oxidization and aggregation. Therefore, the amount of silver loaded, and its subsequent release profile is readily controllable by changing the number of silver-containing layers. The outermost layer is made of an antifouling polymer, poly (sulfobetaine methacrylate-*co*-acrylamide) [poly (SBMA-*co*-AAm)], which also allows free diffusion of silver. Thus, this novel coating is different in having a higher intrinsic silver release capability than the current commercially available silver coated urinary catheters.

Here, we used a previously described mouse model [18, 19] to study the safety and potential efficacy of this catheter against biofilm formation and colonization by UPEC in vivo. To further test the coating for human catheters, we chose a porcine model for our in vivo experiments as it provides a urinary tract that resembles the macroscopic human urinary tract fairly well and is a very accessible animal for laboratory testing [20]. To our knowledge, this model has not been used for a long-term catheterization study. It was also important to compare the efficacy of our catheter to a silver coated catheter currently in use in patients. Therefore, we used an uncoated control in the mouse model whereas a commercially available silver coated catheter control was used in the porcine model.

This study was funded by Technology Enterprise Commercialisation Scheme-Proof-Of-Concept grant of SPRING Singapore (TI/TECS/POC/14/10).

## Methods

### Bacteria and growth conditions

*E. coli* UTI89, an uropathogenic strain known to be a good biofilm developer, isolated from a patient with uncomplicated cystitis, was kindly provided by Dr. Swaine Chen of Genome Institute of Singapore [21]. *E. coli* was cultured in lysogeny broth (LB) (BD Difco, Singapore) at 37 °C with constant agitation at 180–200 rpm. LB agar was used to grow *E. coli* on solid medium and incubated at 37 °C for 12–18 h.

### Catheters for mouse model of CAUTI

Small silicone tubing, SIL037 (3 French, 0.5 mm ID × 0.9 mm OD) was obtained from Braintree Scientific, Inc., USA. Anti-fouling coating (P3) was prepared by coating PDA-AgNP-PDA-AgNP-PDA-poly(SBMA-*co*-AAm) layers on the inside as well as outside surfaces of the silicone tubing as described previously [17]. The method was modified to expedite the coating process as polydopamine was coated with oxygen bubbling at 60 °C for 20 min, instead of in air at room temperature for 24 h in our earlier work. Silver content in the coating was determined to be 33.98 ± 1.62 μg/cm^2^ using Inductive Coupled Plasma-Optical Emission Spectrometry (ICP-OES, iCAP 6200 duo, Thermo Scientific, USA) as previously described [17]. The tubing was cut in 5 mm long segments, autoclaved and air-dried in a clean biosafety cabinet (BSC) before use in mice. Twelve mm long segments of SIL037 catheter tubing were fitted on sterile 25 G needles (Becton Dickinson). A 5 mm long, either coated or uncoated, sterile segment of SIL037 tubing was then placed on top of the 12 mm segment for implantation.

### Catheters for porcine model of CAUTI

Uncoated silicone catheters of 14 Fr size were purchased from Promed Pte. Ltd., Singapore. P3 coated catheter was prepared by coating PDA-AgNP-PDA-AgNP-PDA-poly(SBMA-*co*-AAm) layers on pristine silicone catheter over the interior and exterior of the whole length as described previously with modification to obtain a similar silver content (30.30 ± 1.33 μg/cm^2^) as in the mouse work [17]. For a 14 Fr Foley catheter (40 cm long), total silver amount was estimated to be approximately 3 mg per catheter. A commercially available, Dover™ silver coated 100% silicone Foley catheter was purchased from Covidien Pte. Ltd., Singapore.

Since the balloon inflation port of the silicone catheter was not autoclave-compatible, the coated catheters were disinfected by 70% ethanol followed by UV irradiation for 30 min and sealed before the pig study. The Dover™ catheter was used as received since it was in sterile package.

### Murine model of CAUTI

All animal experiments were conducted in accordance with the university ethical regulations and were approved by the National University of Singapore Institutional Animal Care and Use Committee (IACUC). All animals were allowed an acclimatization period of at least five days in the animal testing facility after arrival, prior to experimentation. Laboratory feed and drinking water were provided ad libitum during entire duration, including before and during experimentation. The animal subjects were provided with environmental enrichment in accordance to species-specific standards. The temperature and humidity of the housing space was regulated as per international standards, and animals were provided with a cycle of 12 h of light followed by 12 h of darkness.

The mouse model was adapted from a previously published study with modifications mainly in the implant size and bacteria used [19]. Briefly, 18–20 weeks old C57BL/6 female mice (from InVivos Pvt. Ltd., Singapore) were anesthetized and 5 mm long catheter pieces were delivered in the urinary bladders by catheterizing the mice with a covered 25 G needle via the urethra lumen. The mice were re-catheterized with IV Catheters (24 G) and 50 μL bacterial cultures (2 x 10^6^ Colony Forming Units (CFU)/ml) were instilled in the urinary bladder. After 30 min dwelling, the bladder was gently pressed to expel the contents.

Urine cultures were collected daily in metabolic cages overnight. Metabolic cages were used so as to collect larger volumes of urine for measurement of silver release from the coating. However, this way of urine collection may increase the risk of contamination of the urine by faeces and feed which we attempted to mitigate by having multiple comparisons. At the end of 14 days, mice were euthanized with CO_2_ overdose (method approved by NUS-IACUC), and catheters were collected (if not passed out in urine). Bacterial biofilm was quantified from the collected catheters with a method using vortexing and sonication as reported recently [22]. In addition, bacterial colonization in the urinary bladder was measured. Each bladder was cut in two halves – one for homogenization and bacterial count, the other for histopathology studies.

### Porcine model of CAUTI

Female miniature pigs (PWG Micropig®) from Prestige BioResearch Pte Ltd., Singapore (PBR) were used for this study. The work was carried out at PBR in accordance with approval by PBR-IACUC. During acclimation, animals were group housed. After the Foley catheter implantation, the animals were individually housed in well labelled steel cages. A standard nutrient and micro-ingredient composition of laboratory animal pig diet ration (pellets - 400 g, Altromin 9029, Altromin Spezialfutter GmbH & Co.KG, Germany) was available to each animal daily. Ultraviolet-irradiated municipal tap water was provided ad libitum to the animals. The animal room environment was controlled: a temperature of 16–27 °C, humidity of 50–80% and approximately 12 h of the light/dark cycle with 150–300 lx.

The study was conducted in compliance with the principles specified in national and international regulatory authority test guideline: ISO 10993:2006 “Biological Evaluation of Medical Devices – Part 11: Tests for Systemic Toxicity”. This guideline largely deals with standardization of biological and clinical methods for evaluation of medical devices [23].

This proof of concept experiment comprised of two animals – one implanted with P3 coated catheter and the other animal with the commercially available silver coated Dover™ catheter for comparison. Implantation of the catheters was performed aseptically under general anaesthesia induced by isoflurane inhalation. The Foley catheter was inserted into the urinary bladder with long surgical forceps via the transurethral route with the animal in dorsal recumbency. The balloon was inflated with 5–6 ml of sterile distilled water according to the manufacturer’s instructions. Subsequently, the inner lumen and outer surface were inoculated with 20 μL each of *E. coli* (10^5^ CFU/ml) in 1X PBS at the outlet. After waiting approximately 10 min for bacteria to attach to the catheter surface, the external part of the catheter was tied to the tail of the pig with suitable adhesives.

Initially a urine reservoir bag was attached to the catheter at all times to maintain a closed system of urine collection as is the case in humans. We were not successful in securing the bag to the animal and observed that the catheter was pulled out or dropped out due to additional pressure from the bag. Therefore, subsequently, this was modified to an open catheter system. The outlet of the Foley catheter was anchored via suturing to skin in a dependent position and the urine bag was used only for urine sample collection.

Animals were euthanized at the endpoint (20 days post inoculation (dpi) for P3 catheter and 21 dpi for Dover™ catheter) by exsanguination of the vena cava and thoracic vein/artery under deep barbiturate anaesthesia (pentobarbitone sodium) as approved by PBR-IACUC. Catheters and organs of urinary tract were collected for evaluation of bacterial colonization and encrustation.

### Assays used for analysis

In mice, efficacy of the coating was analysed using bacterial quantification in urine, on catheters and in tissues, scanning electron microscopy (SEM) and by measuring IL-6 levels in urine. Moreover, safety of the coating was assessed by monitoring body weight, kidney function tests and histopathology examination. Additionally, in the porcine model, encrustation was qualitatively examined visually as well as quantified using ICP-OES to measure calcium and magnesium deposition on catheters and in tissues.

Bacteria in urine and from catheter or tissue samples were counted using Miles and Misra method [24]. SEM was performed as published elsewhere [25] with some modifications and visualised using JEOL JSM-6701F Field Emission Scanning Electron Microscope. IL-6 levels were measured by ELISA to assess the degree of inflammation (Mouse IL-6, Cat# 88–7064-88, eBioscience Inc., USA).

## Results and discussion

### Euthanasia and harvesting

#### Mice

Out of 8 mice implanted with uncoated catheters, two mice were terminated early in the experiment due to bladder outlet obstruction as there was no urine output. An enlarged bladder was observed during post-mortem examination (Additional file 1: Figure S1). Catheters and bladders were collected and processed to assess bacterial colonization. One mouse was terminated 10dpi due to weight loss more than 25% and catheter and organs were collected and processed. The remaining 5 mice were euthanized at the endpoint (14 dpi). All 8 uncoated catheters were recovered.

Among the 8 mice implanted with P3 coated catheters, all mice survived till the endpoint. However, two catheters were lost, possibly, passed with urine from the bladder, hence only 6 catheters were recovered.

#### Pigs

Dover™ catheter in the pig was collected after euthanizing the animal 21 dpi. The P3 catheter did drop out once at two dpi, which was replaced with a new catheter and reinoculated with *E. coli* and the catheter was collected at the endpoint for the animal (20 dpi). It should be noted the pig was implanted with the P3 catheter for 23 days in total.

### Anti-biofilm activity of P3 coating

#### Mice

After the mice were sacrificed, bacterial colonization on the catheter as well as in the bladder was quantified (Table 1). It was found that only one out of four P3 coated catheters had *E. coli* biofilm, whereas five out of six uncoated catheters had developed biofilm. On bacterial quantification, while median values showed a reduction of 10^7^ CFU, average CFU values demonstrated a 50% reduction in bacterial load on P3 coated catheter. Moreover, both uncoated catheters recovered from mice terminated 3–4 dpi due to bladder obstruction were seen to be colonized by high numbers of bacteria (10^7^). The majority of urinary bladders (5 out of 6) implanted with P3 coated catheters did not show any bacterial colonization. In comparison, more than half of the bladders (5 out of 8) implanted with uncoated catheters had bacterial colonization. Moreover, if the two bladders (implanted with uncoated catheters) that were harvested 3–4 dpi were excluded from analysis, the median value of CFU/bladder would be 1.2 X 10^2^ (range: 0 to 6 x 10^7^).

**Table 1:** Catheter recovery, bladder harvesting and *E. coli* colonization

	Uncoated	P3 coated
	Up to 14dpi^a^	14dpi
Number of mice euthanized (total 8 mice per group)	8	8
Catheters collected (catheters lost)	8 (0)	6 (2)
Number of catheters processed for biofilm extraction assay (catheters processed for SEM^b^)	6 (2)	4 (2)
Number of catheters with viable biofilm	5	1
Median values of CFU^c^/catheter (range)	6.58 X 10^7^ (0 to 1.52 X 10^8^)	0 (0 to 1.18 X 10^8^)
Number of urinary bladders collected with catheters	8	6
Number of bladders with viable bacteria	5	1
Median values of CFU/bladder (range)	5.0 X 10^2^ (0 to 1.4 X 10^8^)	0 (0 to 1.03 X 10^5^)

^a^Days post inoculation

^b^Scanning electron microscopy

^c^Colony forming units

**Fig. 1:**
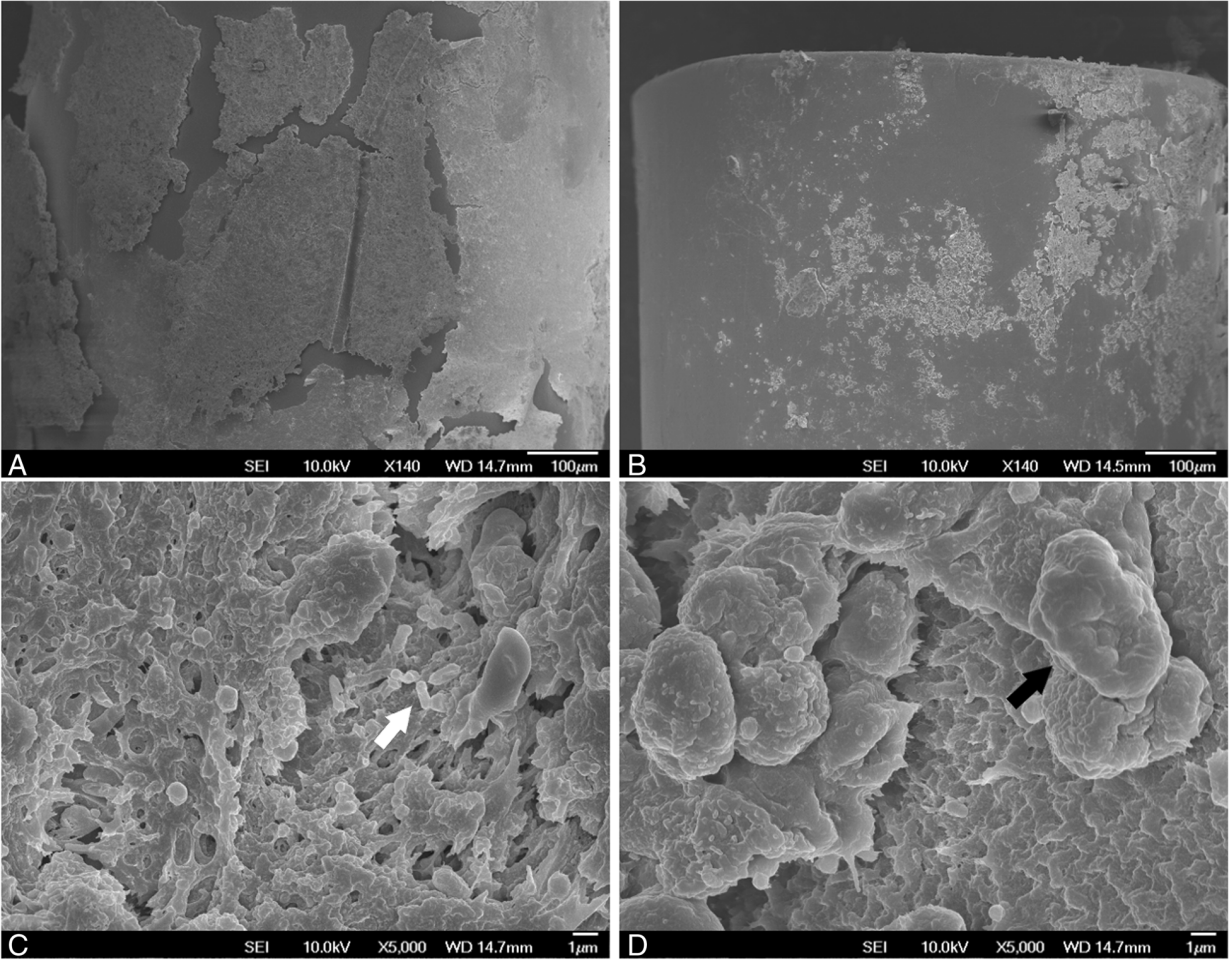
Representative SEM images of differences in biofilm formation on mouse catheters. (**a**) and (**c**) uncoated catheter, (**b**) and (**d**) P3 coated catheter. Scale bars indicate 100 μm for (**a**) and (**b**), and 1 μm for (**c**) and (**d**). The white and black arrows point to structures that suggest bacteria and host cells respectively

Biofilm formation on mouse catheters was visualized by SEM and the differences in coated and uncoated catheters can be seen in Fig. 1. Comparison between uncoated and P3 coated catheters at low magnification in Fig. 1a and b shows the difference in surface area covered with a layer or a film. At high magnification, a few rod-shaped structures resembling *E. coli* shape (and size) can be seen on the uncoated catheter (Fig. 1c – indicated by white arrow), suggesting that these are bacterial biofilms. On the other hand, the size of the cell-like structures seen on P3 coated catheters (Fig. 1d – indicated by black arrow) suggests that they are host cells.

#### Pigs

The human catheter in the porcine model also showed promising results as average bacterial counts from the catheter collected from the pig with the P3 catheter (20 dpi) were 2.4 times lower than that on the silver coated Dover™ catheter (21 dpi). Biofilm extracted from three segments near tip of the catheters recovered from both animals yielded mostly *E. coli* colonies whereas two segments near the urethral exit yielded a polymicrobial biofilm.

**Fig. 2:**
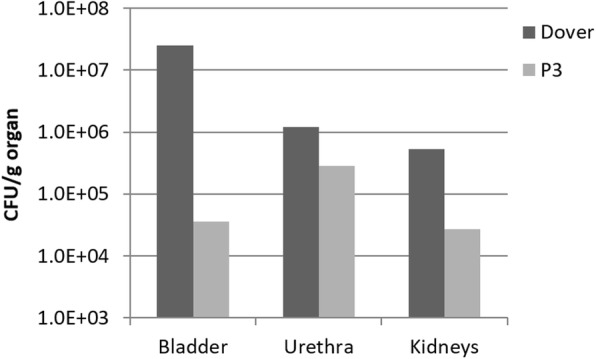
Viable bacterial cell counts from different organs of the micropigs at endpoint

Comparison between *E. coli* inoculated micropigs in Fig. 2 showed a significant (~ 700 fold) reduction in bladder infection with P3 coated catheter as compared to the pig with commercial Dover™ catheter. Similarly, a reduction was also observed in bacterial colonization in the urethra (4 fold) and kidneys (19 fold) (Fig. 2).

Although an uncoated catheter would be an appropriate control to demonstrate efficacy in the pig model, the aim of this study was to develop a catheter which could be marketed for clinical use. However, by comparing the efficacy of our coating to a clinically tested silver coating which is licensed by the major regulatory agencies and in clinical use globally, the results of this proof-of-concept study would be more relevant as various reviews and meta-analyses have demonstrated ability of silver to reduce CAUTI [26–28]. We also do recognise that the proprietary nature of the commercial control catheter limits our ability to compare the details of the different coatings.

These results suggest that the P3 coating could inhibit *E. coli* biofilm formation both on catheter surface and in the bladder effectively in mice and in pigs.

### Antibacterial activity in mice

**Fig. 3:**
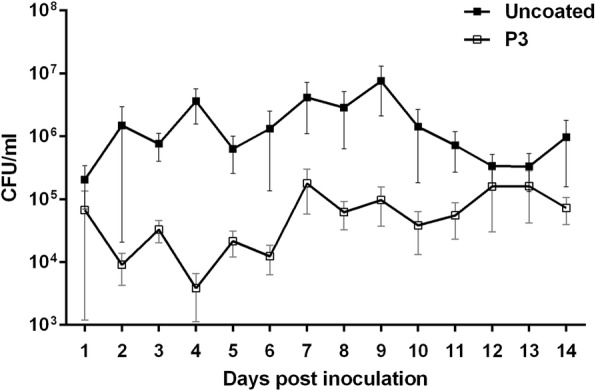
Daily mean bacterial titres in urine of mice inoculated with *E. coli*. Error bars indicate SD

Daily bacterial load in urine was calculated for 14 dpi in six mice implanted with P3 coated catheters. In the uncoated catheter implanted group, bacteriuria was quantified in 5 mice for 14 dpi and one mouse for 10 dpi. Consistently less bacteriuria (51–99%) was observed in mice with P3 coated catheters as compared to mice with uncoated catheters for up to 14 days (Fig. 3).

The higher values of IL6 in uncoated group (Additional file 1: Figure S2) indicating more inflammation as compared to P3 group supporting the finding of low activity of pathogenic bacteria in P3 group mice compared with controls.

### Reduction of encrustation in pigs

**Fig. 4:**
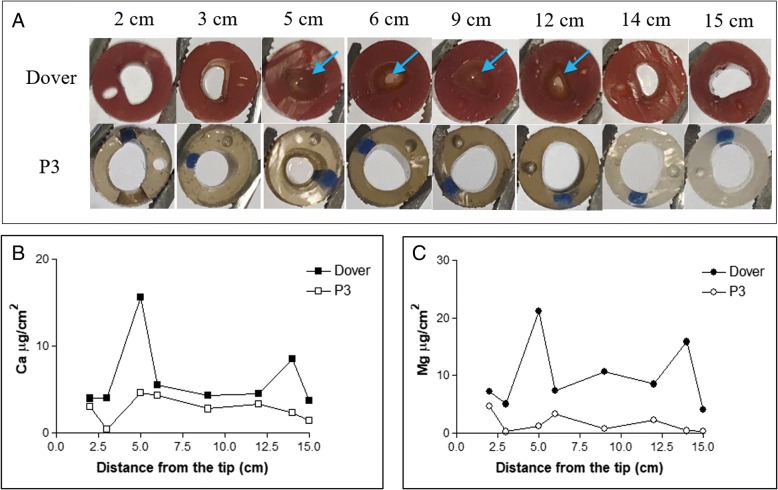
**a**) Photos of cross-section of catheter segments collected from pigs implanted with Dover™ catheter and P3 coated catheter, on 21dpi and 20dpi, respectively. Catheter segments at different positions along the length are shown. Arrows indicate catheter lumen was either partially or completely blocked by crystals and/or biofilm. Average amounts of B) calcium and **c**) magnesium on the catheter segments

Figure 4a shows the cross-sections of the catheters that challenged with *E. coli*. As can be seen, segments of the Dover™ catheter collected 21 dpi were totally or partially blocked, while the P3 coated catheter retrieved 20 dpi remained patent throughout the length assessed (up to 15 cm from the catheter tip, the length inserted in the urethra and bladder). Quantitative results from ICP-OES measurement indicate that both calcium (2.3 times on an average) and magnesium (6 times on an average) amounts were lower on P3 coated catheter as compared to Dover™ catheter (Fig. 4b and c). This indicates that P3 coated catheter inhibited encrustation formation during uropathogenic *E. coli* infection more effectively compared to the commercially available Dover™ catheter.

There were 1.8 times less calcium and magnesium precipitates in the bladder of the pigs with P3 coated catheter as compared to the silver coated Dover™ catheter (Additional file 1: Figure S3).

### Toxicity

All mice lost up to 18% body weight in the first two dpi but recovered well 3rd day onwards (Additional file 1: Figure S4). Mice with the P3 coated catheter show slightly lower (~ 5%) weight loss as compared to mice with uncoated catheters indicating that silver does not appear to cause any general harm to the animals over a period of two weeks.

**Fig. 5:**
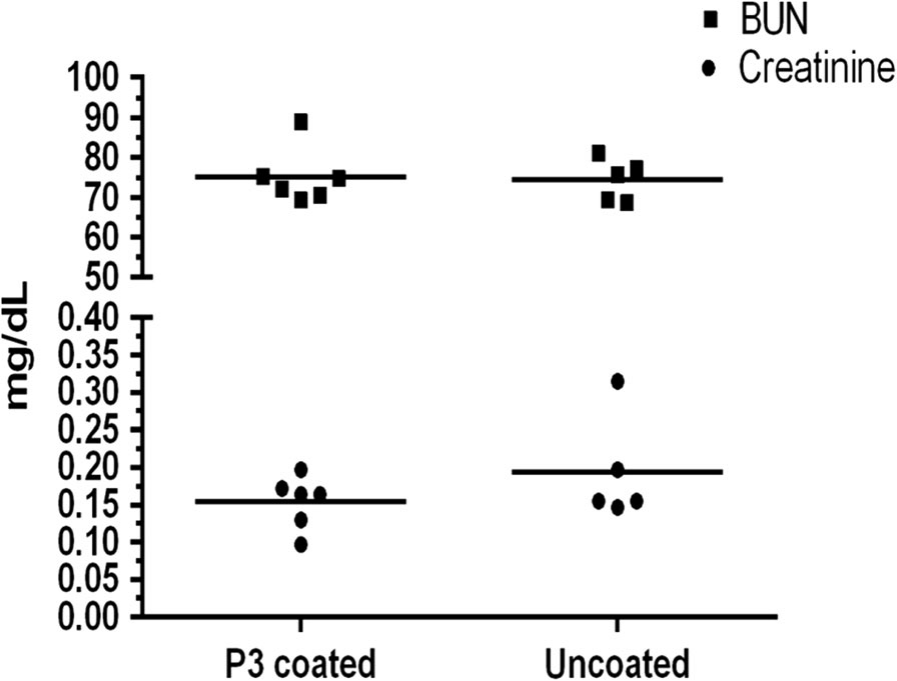
Kidney function tests in C57BL/6 mice inoculated with *E. coli*

Kidney function was also assessed by measuring levels of creatinine and blood urea nitrogen (BUN) in serum at endpoint. As seen in Fig. 5, no major differences were observed in kidney functions (both parameters) of mice with uncoated catheter and those with P3 coated catheters. BUN readings were higher than normal and creatinine values were slightly lower than normal values [29, 30] in both groups indicating that the increase may not be due to P3 coating, but possibly due to the hydration status or inflammatory state of the animals.

Silver levels could not be measured in urine of mice with P3 catheters due to undetectable (low) levels.

### Histopathology

**Fig. 6:**
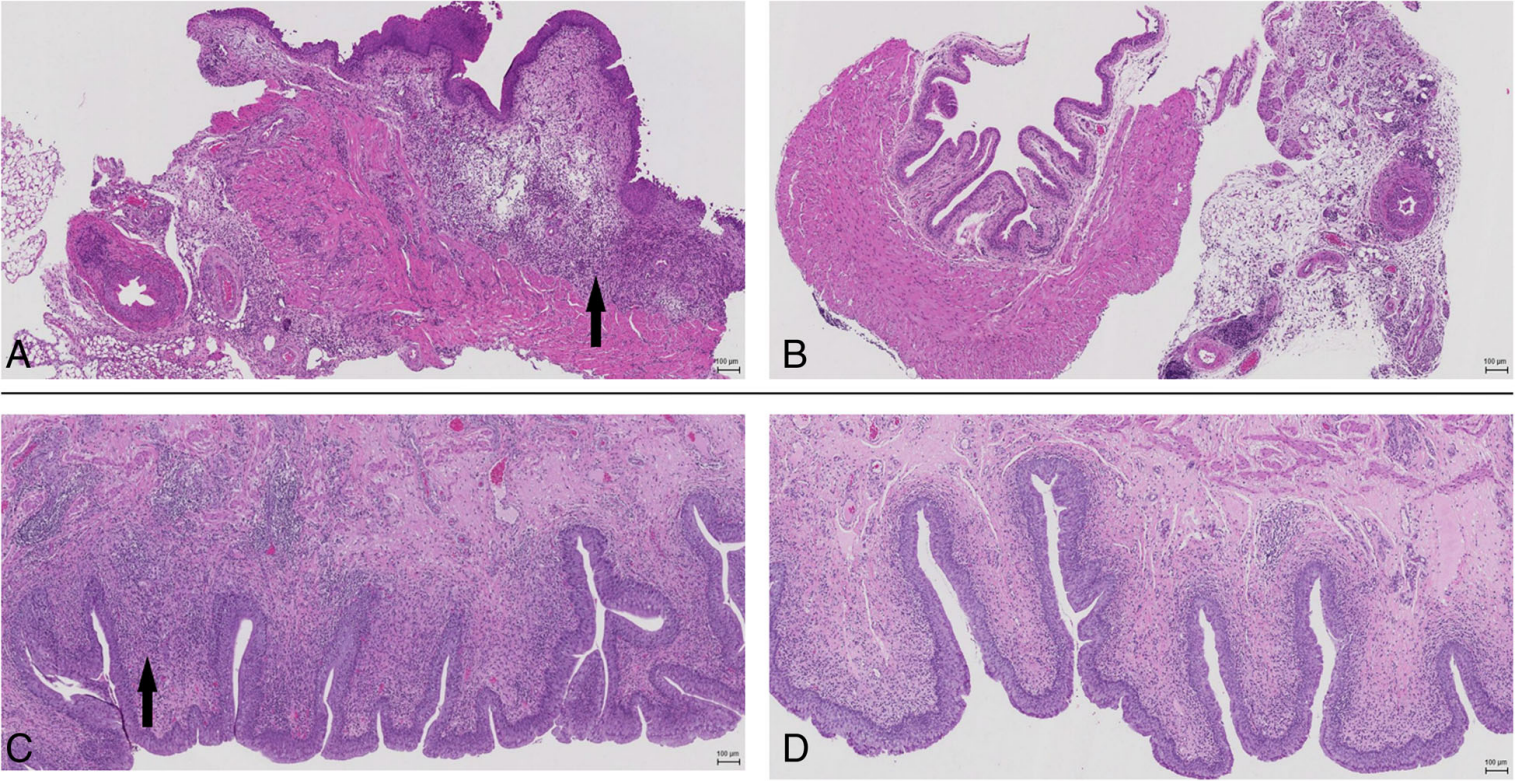
Representative hematoxylin and eosin staining (scale bars 100 μm) of 5 μm section of urinary bladders of **a**) Mice with uncoated catheter and **b**) Mice with P3 coated catheter **c**) Pigs with silver coated Dover™ catheter and **d**) Pigs with P3 coated catheter. Images suggest a higher granulocyte infiltration in the sub-mucosal layer (indicated by black arrows) in **a** and **c** as compared to that in **b** and **d** respectively

The degree of bladder inflammation appeared more severe in the uncoated group as compared to the P3 group. Moderate to severe pyogranulomatous, lymphoplasmacytic, oedematous cystitis as well as minimal to moderate pyogranulomatous, lymphoplasmacytic serositis was seen in mice implanted with uncoated catheters infected with *E. coli* (Fig. 6a)*.* On the other hand, no significant findings were observed in mice with P3-coated catheter and *E. coli* except in one mouse with moderate pyogranulomatous, lymphoplasmacytic serositis (Fig. 6b). Similar results were observed with pig bladders wherein moderate cystitis was seen in animal with the silver coated Dover™ catheter (Fig. 6c) whereas mild cystitis was observed in animal with P3 catheter (Fig. 6d). This indicates that the P3 coating is not harmful to the bladder compared to uncoated catheter (in mice) or silver coated Dover™ catheter (in pigs); it may even have an anti-inflammatory effect.

## Conclusion

In this study, we have demonstrated, using a murine model and a novel porcine model the safety, and antimicrobial effect of the P3 coating. The results also suggest an anti-encrustation capability of P3 coating. In the porcine model, using a proof of concept with a single animal, we demonstrated the potential efficacy of P3 coating in comparison with the commercial Dover™ silver coating. The P3 coated catheter was safe for use as indicated by the absence of systemic clinical and laboratory signs in both animal models. This suggests low toxicity from the catheter coating material in line with ISO 10993–11:2006.

It should be noted that due to the small group size in the study and the limitations of the current pig model (e.g. difficulty in retaining the catheter), more extensive evaluations of the efficacy and safety of the P3 coated catheter are needed before proceeding for regulatory approval, human studies and widespread clinical use of the catheter.

## Additional file

Additional file 1: Supporting information. **Figure S1.** Bladder enlarged due to blockage in urine flow. **Figure S2.** Average IL-6 levels in urine of E. coli inoculated mice implanted with uncoated catheters and P3 coated catheters. Error bars indicate SD. **Figure S3.** Amounts of calcium and magnesium deposition in urinary bladder of all micropigs with DoverTM and P3 catheter. **Figure S4.** Daily weight measurements to assess general health of the mice. Error bars indicate SD. (DOCX 306 kb)

## Abbreviations

[poly(SBMA-*co*-AAm)]: poly(sulfobetaine methacrylate-*co*-acrylamide)
AgNPs: Silver nanoparticles
BSC: Biosafety cabinet
BUN: Blood urea nitrogen
CAUTI: Catheter Associated Urinary Tract Infections
CFU: Colony forming unit
dpi: day(s) post inoculation
IACUC: Institutional Animal Care and Use Committee
ICP-OES: Inductive Coupled Plasma-Optical Emission Spectrometry
LB: Lysogeny broth
P3: Anti-fouling coating prepared by coating PDA-AgNP-PDA-AgNP-PDA-poly(SBMA-*co*-AAm) layers on the inside as well as outside surfaces of the silicone tubing
PBR: Prestige BioResearch Pte Ltd., Singapore
PDA: Polydopamine
SEM: Scanning electron microscopy
UPEC: Uropathogenic *Escherichia coli*
