# Supplementary material for: Restriction of in vivo infection by antifouling coating on urinary catheter with controllable and sustained silver release: a proof of concept study
Source: BMC Infect Dis. 2018 Aug 6;18:370. doi: 10.1186/s12879-018-3296-1 (PMC6090839; doi:10.1186/s12879-018-3296-1)
Supplement: Supplementary file 1 — Supporting information. Figure S1. Bladder enlarged due to blockage in urine flow. Figure S2. Average IL-6 levels in urine of E. coli inoculated mice implanted with uncoated catheters and P3 coated catheters. Error bars indicate SD. Figure S3. Amounts of calcium and magnesium deposition in urinary bladder of all micropigs with DoverTM and P3 catheter. Figure S4. Daily weight measurements to assess general health of the mice. Error bars indicate SD. (DOCX 306 kb) [file 12879_2018_3296_MOESM1_ESM.docx]

Supporting information

Restriction of in vivo infection by antifouling coating on urinary catheter with controllable and sustained silver release: A proof of concept study

*Kedar D. Mandakhalikar, Rong Wang, Juwita N. Rahmat, Edmund Chiong,*

*Koon Gee Neoh, Paul A. Tambyah*

*
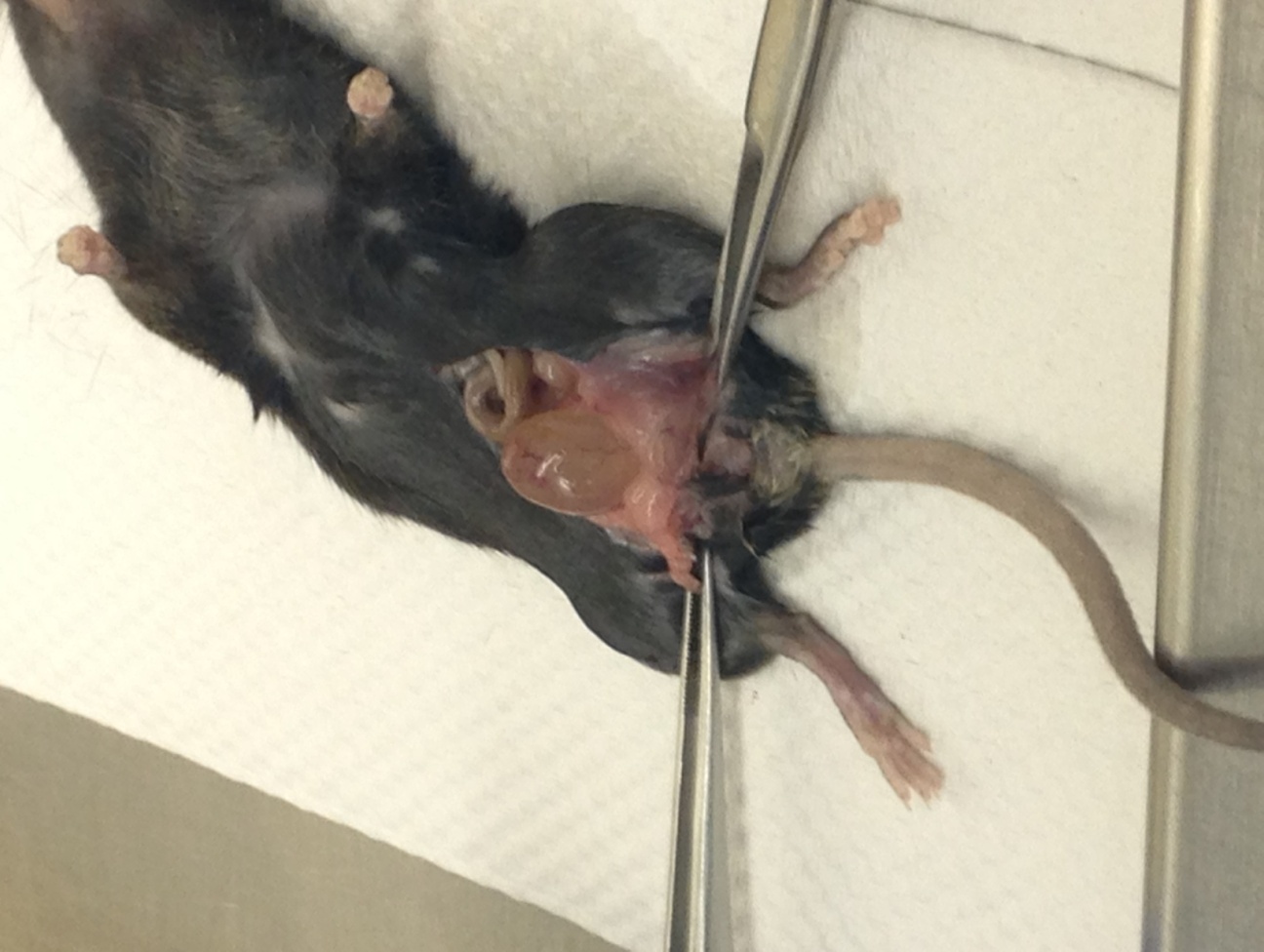
*

Figure S1 Bladder enlarged due to blockage in urine flow

Figure S2 Average IL-6 levels in urine of E. coli inoculated mice implanted with uncoated catheters and P3 coated catheters. Error bars indicate SD.

Figure S3 Amounts of calcium and magnesium deposition in urinary bladder of all micropigs with DoverTM and P3 catheter.

Figure S4 Daily weight measurements to assess general health of the mice. Error bars indicate SD.
